# Supplementary material for: Explaining attractive and repulsive biases in the subjective visual vertical
Source: PLoS Comput Biol. 2026 Apr 1;22(4):e1014159. doi: 10.1371/journal.pcbi.1014159 (PMC13061328; doi:10.1371/journal.pcbi.1014159)
Supplement: Attachment — Submitted filename: Response to Reviewers.pdf [file pcbi.1014159.s001.pdf]

Response to the Editor

We have now added Author Contributions, amended the Financial Disclosure, added the author summary, and published the code as requested.

Concerning the Potential Copyright Issues:

i) Figures 1, and 4 appear to have been adapted from a previously published figure. Please provide written permission from the copyright holder to publish this under our CC-BY 4.0 license, or remove the figure / replace the image. Please note we do not recommend using standard request forms available on Publishers' websites, as they grant single use rather than republication under an open access license.

For Fig. 1: according to ARVO (https://arvojournals.org/ss/terms.aspx): "Authors of IOVS, JOV, and TVST articles retain the right to reuse material from their own articles in their future work, with proper attribution. Permission does not need to be obtained from ARVO." WPM is an author of the cited article.

For Figure 4, permission is not required, since we did not use any part of the original figure that we refer to in the legend. Our figure is completely new and just uses the same way of visually presenting information as the referenced work. We clarify that better now in the legend.

The digitized data and the code to generate the figures are available now at the German G-Node repository (https://doi.org/10.12751/g-node.k52s4k).

Response to reviewers

We thank both reviewers for their positive evaluation.

We have changed various small details, most importantly we now express variability as circular standard deviation, which for small angles corresponds to linear standard deviation, while in our last version it was expressed as square root of circular variance. Additional improvements include a numerically more stable implementation of the von Mises distribution (see code) which improved the fitting to the data, and additional model variants with different priors.

In the following, we provide point-by-point answers to all questions and comments.

Reviewer 1

As the authors point out, repulsive biases only arise in the model if the posterior mean is used. While this estimator is appropriate for modelling performance on a continuous adjustment SVV, does it raise the possibility that different task structures (e.g. binary choice, is line tilted towards/away from body relative to vertical) might yield different results?

This is an excellent suggestion. In many SVV experiments, a 2AFC task is used asking for a binary decision. In this case, the cost or loss function no longer depends on the size of error being made. For 2AFC, there is one of two possible responses; for example, “CW” if the indicator line is tilted clockwise with respect to the perceived vertical, or “CCW” if perceived tilted in the counterclockwise direction. In the psychometric analysis of such responses, the point of subjective equality (PSE) is the value reported as SVV. A Bayesian observer reports “CW” for all line orientations for which the probability of a clockwise deviation is larger than 0.5. The PSE thus corresponds to the so-called “circular half-mass split” median of the posterior density. The difference to the circular mean is not very large in our model but leads to a slightly smaller E-effect than in an adjustment task.
The variability reported in a 2AFC task is related to the slope at the PSE, which for a Gaussian posterior is proportional to the inverse of the standard deviation. However, in our model, the posterior is non-Gaussian and asymmetric, therefore the variability depends on the local density at the decision boundary. The variability for 2AFC is, according to the model, for tilts <60° smaller than for adjustment, but larger for larger head tilts.
We added a paragraph to the results and the new Fig. 9 to show the difference in results for different task structures (adjustment versus 2AFC).

Being able to account both for situations in which repulsive biases do and do not occur would add weight to the proposed explanation.

Done. As we show in Fig.8, the repulsive bias (8A) depends on the signal dependence of the angular noise (8B), which in turn depends in our model on the relation between utricular and saccular noise (8C). Thus, reducing uncertainty in measuring acceleration along the head z-axis could in principle also reduce the repulsive bias.
We added one sentence at page 12.

How robust are modelling outcomes to the introduction of non-Gaussian otolith noise? Are asymmetric likelihoods and repulsive biases retained under more realistic neural noise statistics?

Gaussian otolith noise is a common assumption (e.g. Tarnutzer et al. 2009), which is also justified by the larger number of afferent fibers involved: according to Curthoys (2020) in humans there are “around 33,000 receptors in each utricular macula (synapsing on around 6,000 afferents) and 18,000 receptors in each saccular macula (synapsing on around 4,000 saccular afferents)”.
However, the firing rate of a single afferent fiber is certainly better described by a non-Gaussian distribution, e.g. lognormal. Nonetheless, for a combination of several thousand afferent fibers, combined via commissural and cross-striolar inhibitory connections, the resulting distribution would be close to Gaussian, especially because the coefficient of variation (CV) of regular otolith units has been reported to be relatively low (<0.075 according to Fernandez & Goldberg 1976).

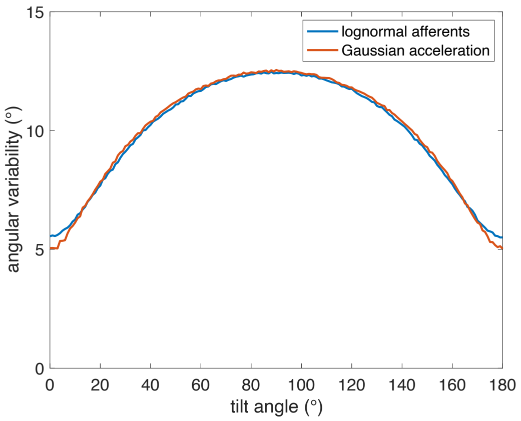

Figure 1: Variability of measured tilt angle (square root of angular variance) for lognormal afferents (blue) and acceleration with Gaussian noise (red). Both curves have been generated by numerical Monte-Carlo simulation (see text).

To evaluate the effect of using lognormal distributions for afferent activation, we used Monte-Carlo simulation (n=100000 samples). Acceleration responses were calculated from inhibitory combination of two lognormal distributions (mean firing rate 40 Hz, amplitude at +1g 60 Hz, at -1g 20 Hz) corresponding to two oppositely oriented hair cell populations, and the resulting angle was calculated via arctangent. We then adjusted the CVs of the utricular (CV 0.075) and saccular (CV 0.23) lognormal distributions to match the angular variability that resulted from using normally distributed noise as in Eqn. 5. For the Gaussian acceleration noise, we used the parameters fitted to the data in the manuscript. The resulting angular variability matched extremely well (Figure 1 above).
We conclude that using a more realistic lognormal distribution of afferent firing rates, combined by cross-striolar or commissural inhibition, leads to a similar result concerning the stimulus-dependent variability of the measured angle.

Two sentences mentioning this result (without showing details) have been added to the Discussion at page 16.

Minor points:

P1. 'in the upright position, the [mean] SVV error is close to zero'

Done.

Figure 2. At printed scale, the repulsive E-effect is not clearly visible as stated in the legend.

Done. We added an inset to Fig. 2B that shows the relatively small E-effect occurring with the default parameters of the idiotropic model.

Reviewer #2: The authors present a Bayesian model of attractive and repulsive bias during a specific perceptual inference task. Although other models of the behavioural pattern already exist, theirs is the first normative (Bayesian) model to jointly account for both the attractive and repulsive biases with minimal additional assumptions.

I found the manuscript convincing, employing a principled approach to a well-defined problem. I have no technical concerns regarding the provided models. I do think the manuscript could benefit from more explicit contextualization and presentation of relevant information (described in detail below), but this is overall minor.

In particular, I think it would suit the authors to clarify the following:
- Although the task itself is well-presented, not much motivation is provided as to why the task is particularly notable in the broad setting of perceptual inference tasks. (Is it just that it yields both attractive and repulsive biases? Is there anything else?)

Done. We have expanded on the motivation in the revised text. One reason is indeed that both types of biases are found, with the repulsive bias being present only sometimes, which is another unusual finding that in our model is explained by the relative uncertainty of the otolith receptors. Another reason is that the SVV belongs to the earliest historical descriptions of perceptual distortions: Aubert's paper was published 1861, the Zöllner illusion and the Poggendorff illusion 1860, the Hering illusion 1861, the Vierordt effect in time perception 1868, the Müller-Lyer illusion also 1868. Furthermore, in contrast to many other perceptual tasks, the SVV is not only of interest for Experimental Psychology or Neuroscience of Perception but has an importance in neurology and clinical diagnosis of vestibular disorders. Taken together, we think that this provides sufficient motivation for our work.

- Interpreting the model fits requires identifying the signatures of the A- and E-effects on plots like the ones in Figure 2. I found that doing this took some effort. I suggest that, given how important visual interpretation of these effects is, the authors find a visual way of demonstrating or illustrating them (e.g., via highlighting or by an additional panel with schematized plots)

Done. We added an inset to Fig. 2B to show the E-effect of the idiotropic model and added a sentence to explain the A-effect.

- It might be beneficial to have a two-sentence primer on the biology early in the paper (it is possible to pick this up incidentally, but it would have made for a smoother reading experience to have it earlier)

Done. We added one sentence about the SVV and the otoliths in the introduction together with some new references.

- What's the point of fitting the Bayesian models to the simulated outputs from the idiotropic model when you later fit to participant data (which is presumably what actually matters)?

We understand the reviewer's point; we could indeed have used data from the literature right from the beginning. However, we prefer to stay with the model comparison for the following reason: the idiotropic model has been shown multiple times to offer an excellent description of average SVV results in humans with very few parameters and therefore constitutes a baseline for comparison with other models. Any other model

should therefore be able to replicate the simulation results of the idiotropic model. A direct comparison of new results to the idiotropic model is therefore useful to judge whether the new model will be able to simulate real data as well.

- It may be worth explicitly distinguishing between "measured value" (meaning, the output of the vestibular sensors) and "observed value" (meaning, what the participants actually reported) — since these are both, in a sense, "measured", some might be confused by this. This is especially relevant in Figure 7 where "measured", as far as I can tell, actually is meant in the latter sense whereas generally throughout the article it is mostly meant in the former sense.

Done. In Fig. 7, the measured value is the one that results from the sensory measurement of the vestibular sensors (thin black line). The perceived (or reported) value in Fig. 7 is the thick dashed black line, the mean of the posterior distribution.
To avoid confusion, we have replaced "measured" by "assessed" in the abstract and on page 9 ("assessed the SVV" instead of "measured the SVV"). Thus "measured" now always refers to the sensory measurement.

- In figure 4 it may be worth annotating what each entry of each matrix encodes. For example, each entry of the prior is P(theta), each entry of the likelihood matrix is P(measurement | tilt angle), and each entry of the posterior matrix is P(SVV estimate | measurement)

Done. To avoid clutter in the graphics, we added the respective probability densities in the figure caption. We hope that this makes it clearer.

- Since the response variability estimated by your model (i.e., based on the posterior dispersion) is systematically higher than observed response variability, it may be worth a discussion of elements that may account for this potentially-significant difference.

The larger response variability predicted by the model was indeed puzzling. In our previous version, we fitted the model to the average SVV settings only and *predicted* the variability, i.e., the model's response variability shown in Fig. 5B (red) depended on the exact shape of the data in Fig. 5A.
We now took a different approach and simultaneously fitted the SVV adjustments and the SVV response variability. We also verified that we used the same approach as Van Beuzekom & Van Gisbergen, who estimated the average within-subject variability. Our new fit of the full-likelihood model with von-Mises prior is shown in Fig. 5. The response variability, expressed as circular standard deviation, now matches the data quite well. It is smaller for very small and very large angles, but larger for intermediate ones.
Following previous work (Willemsen et al. 2022), we also fitted model variants that used different priors than the von-Mises prior (see Methods, last part). The model fit using a t location-scale prior is shown in Fig. 10, it provides a much closer fit to the variability but doesn't show a clear E-effect. However, also this model can still show an E-effect if the difference between utricular and saccular noise becomes sufficiently large. We finally show that the differences between the fitted estimation models do not have major consequences for stimuli which are generated by non-matching generative models, which points towards a certain robustness of the estimation process.
